# Dr. Isaac Girard Wheeler

**Published:** 1906-06

**Authors:** 


DR. ISAAC GIRARD WHEELER, of Buffalo, died at his home in this city, May 22, 1906, aged about 72 years. He was a graduate of Albany Medical College, 1874, and had practised in Buffalo many years.

